# Supplementary material for: Soil net nitrogen mineralisation across global grasslands
Source: Nat Commun. 2019 Oct 31;10:4981. doi: 10.1038/s41467-019-12948-2 (PMC6823350; doi:10.1038/s41467-019-12948-2)
Supplement: Supplementary file 2 — Supplementary Information [file 41467_2019_12948_MOESM2_ESM.pdf]

Supplementary Information

Soil net nitrogen mineralisation across global grasslands

Risch et. al

Supplementary Figures

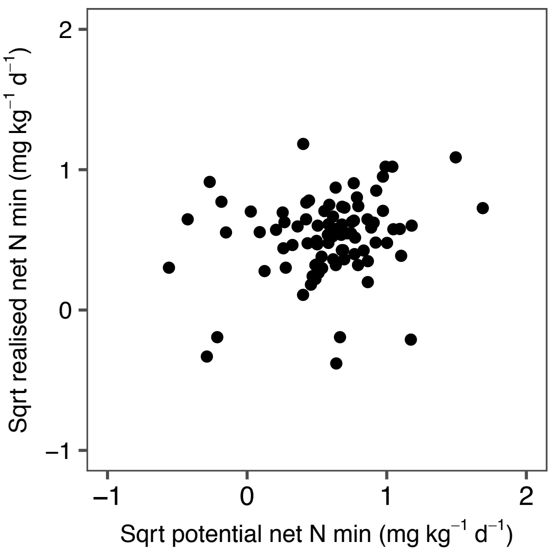

Supplementary Figure 1. Relationship between realised and potential soil net N mineralisation (soil net N_{min}). Points represent the values for individual plots. Results of statistical analyses can be found in the main text and are based on Pearson correlation. Note that the values for realised and potential soil net N_{min} were square root (sqrt)-transformed. Total number of observations = 85. Source data are provided in the source data file.

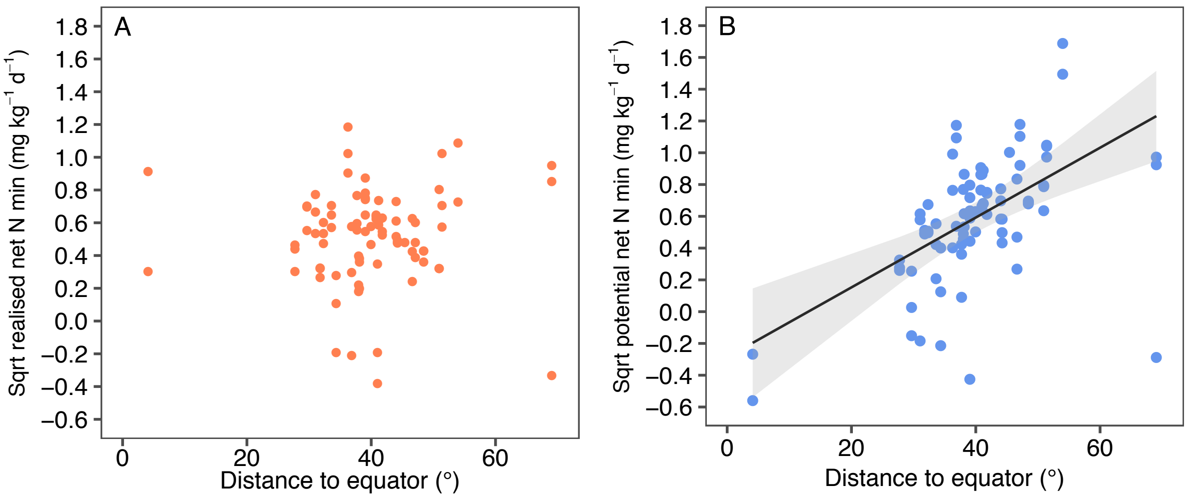

Supplementary Figure 2. Global spatial patterns in realised and potential soil net N mineralisation (soil net N_{min}). (A) Relationship of realised soil net N_{min} with increasing distance to the equator. (B) Relationship of potential soil net N_{min} with increasing distance to the equator. Results of statistical analyses can be found in the main text and are based on linear mixed effect models with site identity included as a random factor. Note that the values for realised and potential soil net N_{min} were square root (sqrt)-transformed. Points represent the values for individual plots. Distance to the equator represents absolute latitude.Total number of observations = 85. Source data are provided in the source data file.

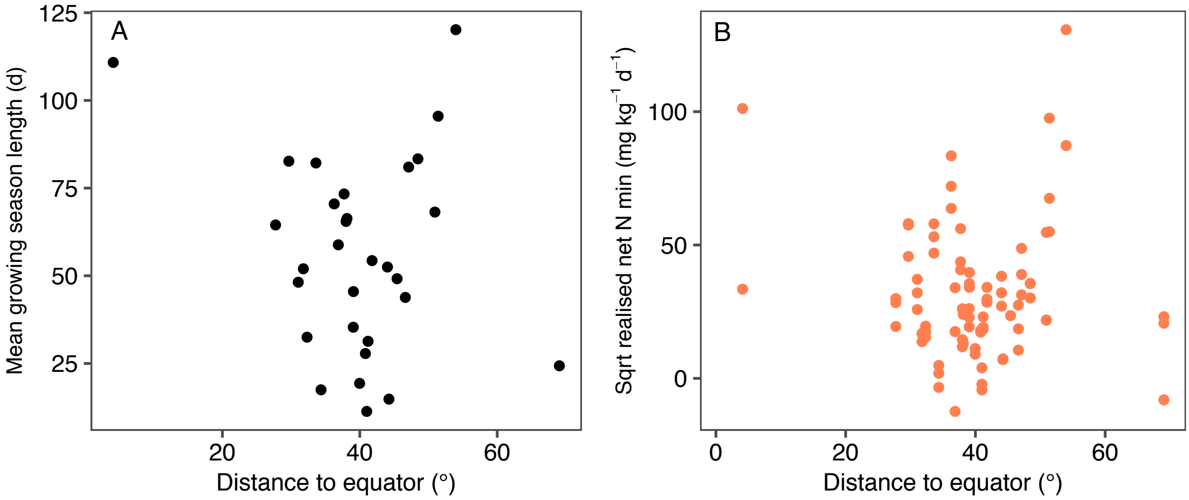

Supplementary Figure 3. Realised soil net N mineralisation corrected for growing season length. (A) growing season length for each site (*n* = 30), (B) Realised soil net N mineralisation estimated for the entire growing season, realised soil net N_{min} values were square root (sqrt)-transformed. Distance to the equator represents absolute latitude. Points represent the individual values for each realised soil net N_{min} value at each site (*n* = 85). Source data are provided in the source data file.

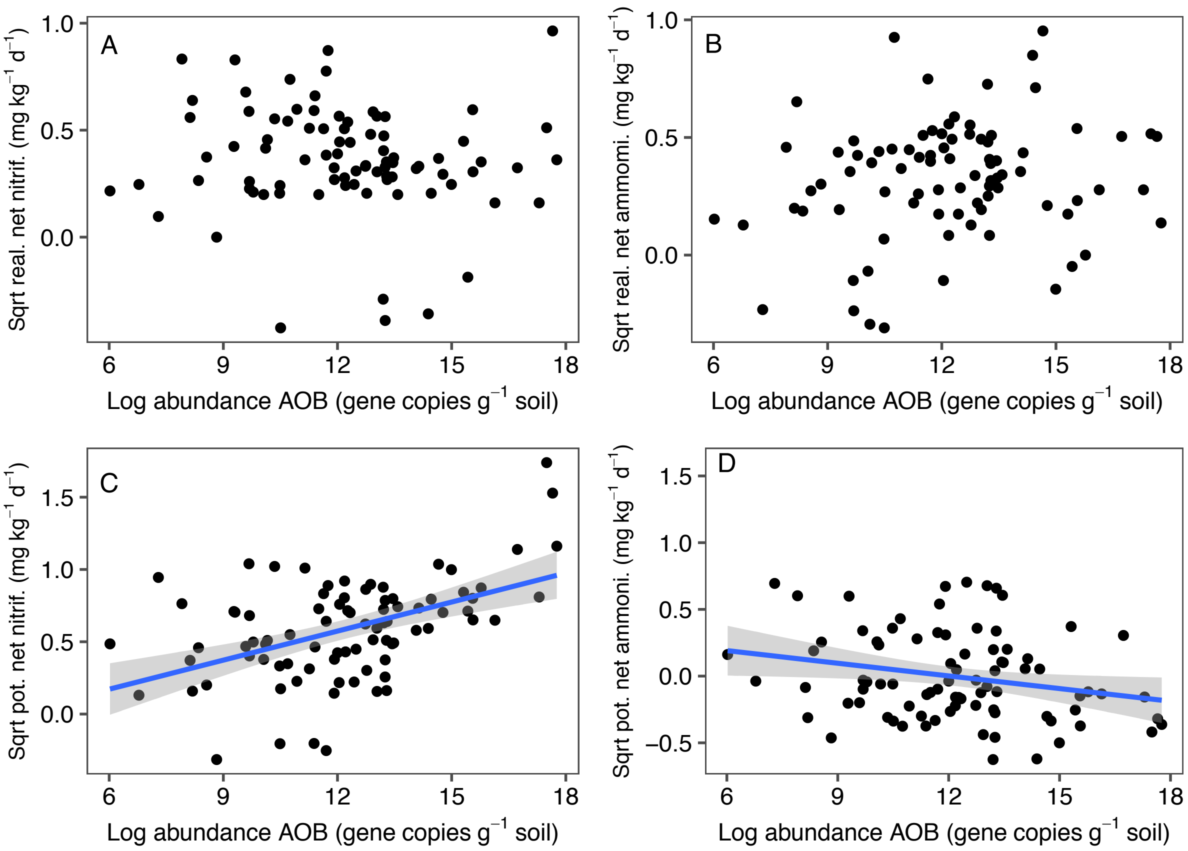

Supplementary Figure 4. Relationship between the abundance of ammonia oxidising bacteria (AOB) and soil net nitrification and ammonification. A) realised and C) potential soil net nitrification, B) realised and D) potential soil net ammonification. Note that realised and potential soil net nitrification and ammonification values were square root (sqrt)-transformed, the abundance of AOB was ln (natural log) transformed. Total number of observations = 85. real. = realised, pot. = potential, nitrif. = nitrification, ammoni. = ammonification, AOB = ammonia oxidizing bacteria. Source data are provided in the source data file.

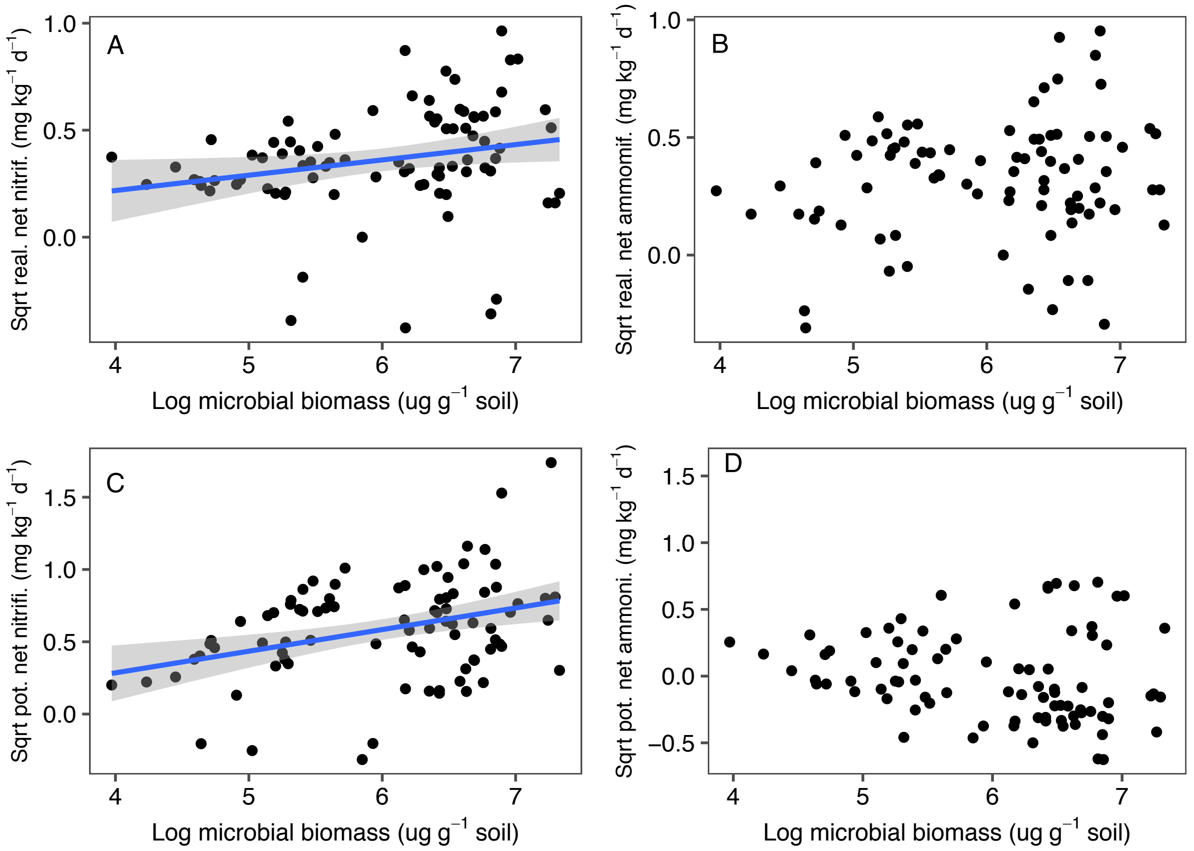

Supplementary Figure 5. Relationship between microbial biomass and soil net nitrification and ammonification. A) realised and C) potential soil net nitrification, B) potential and D) realised soil net ammonification. Note that realised and potential soil net nitrification and ammonification values were square root (sqrt)-transformed, microbial biomass was ln (natural log) transformed. Total number of observations = 85. real. = realised, pot. = potential, nitrif. = nitrification, ammoni. = ammonification. Source data are provided in the source data file.

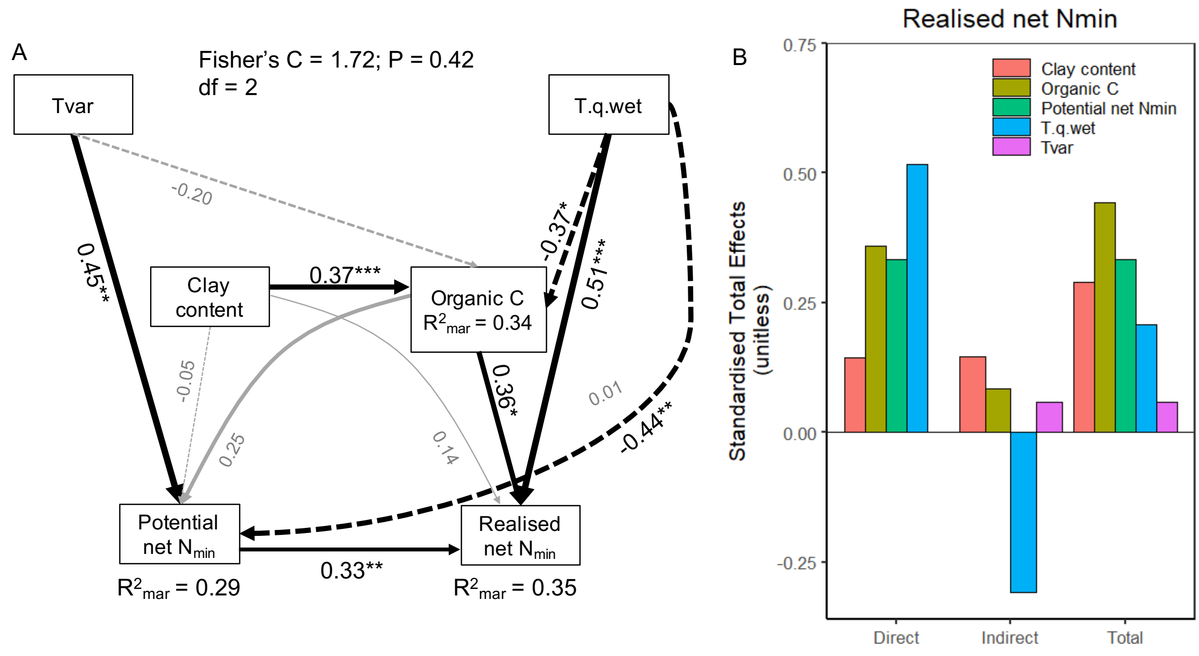

Supplementary Figure 6. Global drivers of realised soil net N mineralisation (soil net N_{min}). (A) Structural equation modelling diagram representing connections between climatic conditions, soil physical, chemical and biological properties found to influence realised and potential soil net N mineralisation. Note that in this model microbial biomass was replaced with soil organic C. The width of the connections represents estimates of the standardised path coefficients, with solid lines representing a positive relationship and dashed lines a negative relationship. Significant connections and R^2 are shown in black, non-significant ones in light-grey. (B) Standardised total, direct and indirect effects of variables associated with realised soil net N min. †$p < 0.1$, *$p < 0.05$, **$p < 0.01$, ***$p < 0.001$. Clay content = soil clay content, Organic C = soil organic C, Tvar = temperature seasonality, T.q.wet = temperature of the wettest quarter. The total number of observations = 85, the total number of sites = 30. Source data are provided in the source data file.

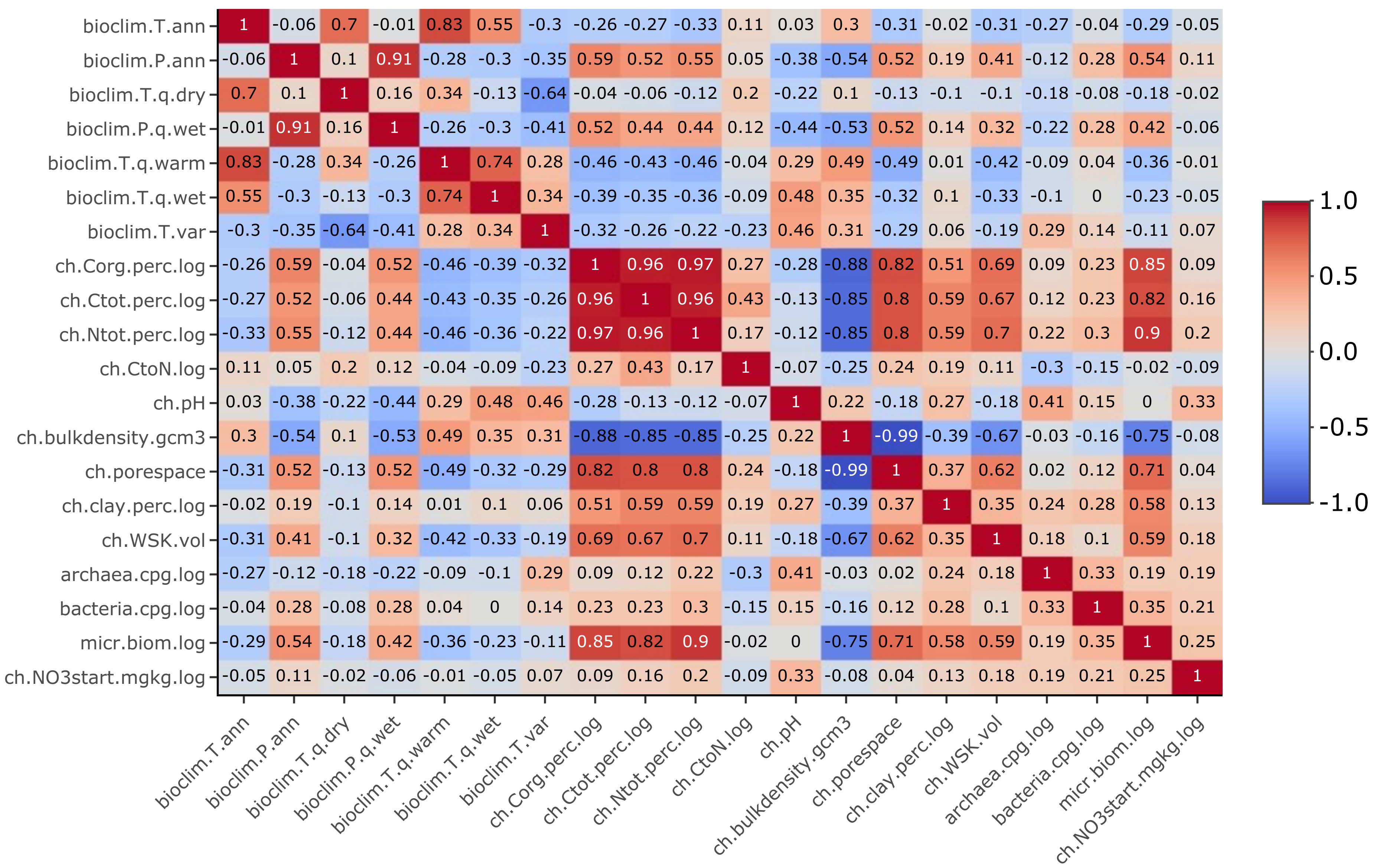
bioclim.T.ann 1 -0.06 0.7 -0.01 0.83 0.55 -0.3 -0.26 -0.27 -0.33 0.11 0.03 0.3 -0.31 -0.02 -0.31 -0.27 -0.04 -0.29 -0.05
bioclim.P.ann -0.06 1 0.1 0.91 -0.28 -0.3 -0.35 0.59 0.52 0.55 0.05 -0.38 -0.54 0.52 0.19 0.41 -0.12 0.28 0.54 0.11
bioclim.T.q.dry 0.7 0.1 1 0.16 0.34 -0.13 -0.64 -0.04 -0.06 -0.12 0.2 -0.22 0.1 -0.13 -0.1 -0.1 -0.18 -0.08 -0.18 -0.02
bioclim.P.q.wet -0.01 0.91 0.16 1 -0.26 -0.3 -0.41 0.52 0.44 0.44 0.12 -0.44 -0.53 0.52 0.14 0.32 -0.22 0.28 0.42 -0.06
bioclim.T.q.warm 0.83 -0.28 0.34 -0.26 1 0.74 0.28 -0.46 -0.43 -0.46 -0.04 0.29 0.49 -0.49 0.01 -0.42 -0.09 0.04 -0.36 -0.01
bioclim.T.q.wet 0.55 -0.3 -0.13 -0.3 0.74 1 0.34 -0.39 -0.35 -0.36 -0.09 0.48 0.35 -0.32 0.1 -0.33 -0.1 0 -0.23 -0.05
bioclim.T.var -0.3 -0.35 -0.64 -0.41 0.28 0.34 1 -0.32 -0.26 -0.22 -0.23 0.46 0.31 -0.29 0.06 -0.19 0.29 0.14 -0.11 0.07
ch.Corg.perc.log -0.26 0.59 -0.04 0.52 -0.46 -0.39 -0.32 1 0.96 0.97 0.27 -0.28 -0.88 0.82 0.51 0.69 0.09 0.23 0.85 0.09
ch.Ctot.perc.log -0.27 0.52 -0.06 0.44 -0.43 -0.35 -0.26 0.96 1 0.96 0.43 -0.13 -0.85 0.8 0.59 0.67 0.12 0.23 0.82 0.16
ch.Ntot.perc.log -0.33 0.55 -0.12 0.44 -0.46 -0.36 -0.22 0.97 0.96 1 0.17 -0.12 -0.85 0.8 0.59 0.7 0.22 0.3 0.9 0.2
ch.CtoN.log 0.11 0.05 0.2 0.12 -0.04 -0.09 -0.23 0.27 0.43 0.17 1 -0.07 -0.25 0.24 0.19 0.11 -0.3 -0.15 -0.02 -0.09
ch.pH 0.03 -0.38 -0.22 -0.44 0.29 0.48 0.46 -0.28 -0.13 -0.12 -0.07 1 0.22 -0.18 0.27 -0.18 0.41 0.15 0 0.33
ch.bulkdensity.gcm3 0.3 -0.54 0.1 -0.53 0.49 0.35 0.31 -0.88 -0.85 -0.85 -0.25 0.22 1 -0.99 -0.39 -0.67 -0.03 -0.16 -0.75 -0.08
ch.porespace -0.31 0.52 -0.13 0.52 -0.49 -0.32 -0.29 0.82 0.8 0.8 0.24 -0.18 -0.99 1 0.37 0.62 0.02 0.12 0.71 0.04
ch.clay.perc.log -0.02 0.19 -0.1 0.14 0.01 0.1 0.06 0.51 0.59 0.59 0.19 0.27 -0.39 0.37 1 0.35 0.24 0.28 0.58 0.13
ch.WSK.vol -0.31 0.41 -0.1 0.32 -0.42 -0.33 -0.19 0.69 0.67 0.7 0.11 -0.18 -0.67 0.62 0.35 1 0.18 0.1 0.59 0.18
archaea.cpg.log -0.27 -0.12 -0.18 -0.22 -0.09 -0.1 0.29 0.09 0.12 0.22 -0.3 0.41 -0.03 0.02 0.24 0.18 1 0.33 0.19 0.19
bacteria.cpg.log -0.04 0.28 -0.08 0.28 0.04 0 0.14 0.23 0.23 0.3 -0.15 0.15 -0.16 0.12 0.28 0.1 0.33 1 0.35 0.21
micr.biom.log -0.29 0.54 -0.18 0.42 -0.36 -0.23 -0.11 0.85 0.82 0.9 -0.02 0 -0.75 0.71 0.58 0.59 0.19 0.35 1 0.25
ch.NO3start.mgkg.log -0.05 0.11 -0.02 -0.06 -0.01 -0.05 0.07 0.09 0.16 0.2 -0.09 0.33 -0.08 0.04 0.13 0.18 0.19 0.21 0.25 1
bioclim.T.ann
bioclim.P.ann
bioclim.T.q.dry
bioclim.P.q.wet
bioclim.T.q.warm
bioclim.T.q.wet
bioclim.T.var
ch.Corg.perc.log
ch.Ctot.perc.log
ch.Ntot.perc.log
ch.CtoN.log
ch.pH
ch.bulkdensity.gcm3
ch.porespace
ch.clay.perc.log
ch.WSK.vol
archaea.cpg.log
bacteria.cpg.log
micr.biom.log
ch.NO3start.mgkg.log
1.0
0.5
0.0
-0.5
-1.0

Supplementary Figure 7. Correlations between all predictor variables prior to variable selection (Supplementary Table 4). Pearson correlation coefficients for all pairs of variables were calculated with the R function 'cor' and the correlogram displayed using the R library "heatmaply". Bioclim.T.ann = mean annual temperature, bioclim.P.ann = mean annual precipitation, bioclim.T.q.dry = temperature of the driest quarter, bioclim.P.q.wet = Precipitation of the wettest quarter, bioclim.T.q.warm = temperature warmest quarter, bioclim.T.q.wet = temperature wettest quarter, bioclim.T.var = temperature seasonality, ch.Corg.perc.log = soil organic carbon, ch.Ctot.perc.log = soil total carbon, ch.Ntot.perc.log = soil total nitrogen, ch.CtoN = soil carbon:nitrogen ratio, ch.pH = soil pH, ch.bulkdensity.gcm3 = soil bulk density, ch.porespace = soil pore space, ch.clay.perc.log = soil clay content, ch.WSK.vol = soil water holding capacity, archaea.cpg.log = amount of ammonia oxidising archaea g^{-1} soil, bacteria.cpg.log = amount of ammonia oxidising bacteria g^{-1} soil, micr.biom.log = microbial biomass, ch.NO3start.mgkg.log = NO_3- content at the start of the incubation. Several variables were log-transformed due to their skewed distribution. Correlated variables r > |0.7|), but for the three variables soil bulk density-soil organic C-microbial biomass were removed for statistical analyses. Final variable selection and their correlated counterparts can be found in Supplementary Table 5. Source data are provided in the source data file.

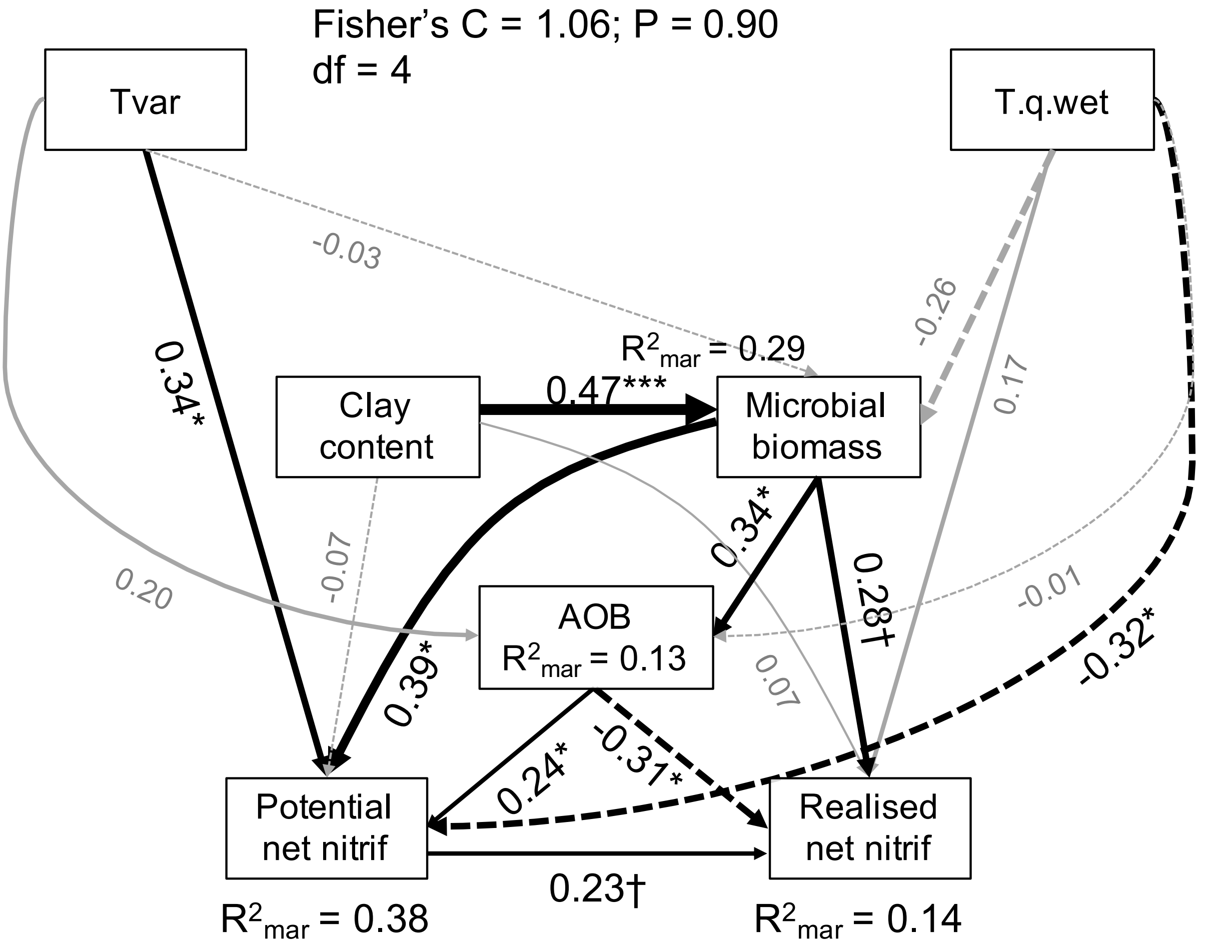

Supplementary Figure 8. Global drivers of realised soil net nitrification. Structural equation modelling diagram representing connections between climatic conditions, soil physical, chemical and biological properties found to influence realised and potential soil net nitrification. *Note that in this model we included links between AOB and potential as well as AOB and realised soil net nitrification.* The width of the connections represents estimates of the standardised path coefficients, with solid lines representing a positive relationship and dashed lines a negative relationship. Significant connections and R^2 are shown in black, non-significant ones in light-grey. AOB = ammonia oxidising bacteria, Clay content = soil clay content, Organic C = soil organic C, Tvar = temperature seasonality, T.q.wet = temperature of the wettest quarter. The total number of observations = 85, the total number of sites = 30. nitrif = nitrification. Source data are provided in the source data file.

Supplementary tables

Supplementary Table 1. Site, continent of site location, country of site location, grassland type, elevation, latitude (in °), longitude (in °), mean annual temperature (MAT, in °C), mean annual precipitation (MAP, in mm) and principal investigator(s) of the 30 Nutrient Network sites included in this study. Descriptions of the range of soil edaphic conditions at our sites can be found within the main text and Supplementary Table 2. AR = Argentina, US = United States of America, AU = Australia, PT = Portugal, CA = Canada, CH = Switzerland, DE = Germany, IN = India, FI = Finland, UK = United Kingdom, EC = Ecuador, ZA = South Africa.

Site	Continent	Country	Grassland type	Elevation	Latitude	Longitude	MAT	MAP	Site PI
bari.ar	South America	AR	grassland steppe	786	-41.01	-71.15	8.6	862	Speziale/di Virgilio
bldr.us	North America	US	shortgrass prairie	1633	39.97	-105.23	9.7	425	Davis/Melbourne
bogong.au	Australia	AU	alpine grassland	1760	-36.87	147.25	5.7	1592	Moore/Morgan
burrawan.au	Australia	AU	semiarid grassland	425	-27.73	151.14	18.4	683	Firn/Buckley
cbgb.us	North America	US	tallgrass prairie	275	41.79	-93.39	9	855	Biedermann/Harpole
cdcr.us	North America	US	tallgrass prairie	270	45.43	-93.21	6.3	750	Borer/Seabloom
cdpt.us	North America	US	shortgrass prairie	965	41.20	-101.63	9.5	445	Knops
chilcas.ar	South America	AR	mesic grassland	15	-36.28	-58.27	15.1	925	Yahdijan/Chaneton/Tognetti
comp.pt	Europe	PT	annual grassland	200	38.82	-8.79	16.5	554	Caldeira/Bugalho
cowi.ca	North America	CA	old field	50	48.46	-123.38	9.8	764	MacDougall
frue.ch	Europe	CH	pasture	995	47.11	8.54	6.5	1355	Hautier/Guesewell
jena.de	Europe	DE	grassland	320	50.93	11.53	8	610	Ebeling/Roscher
kibber.in	Asia	IN	alpine grassland	4241	32.32	78.01	1.1	504	Sankaran
kilp.fi	Europe	FI	tundra grassland	700	69.05	20.83	-4.1	551	Eskelinen/Virtanen
koffler.ca	North America	CA	pasture	301	44.02	-79.54	6.4	815	Cadotte
konz.us	North America	US	tallgrass prairie	440	39.07	-96.58	11.9	877	Blair/Smith/La Pierre
lancaster.uk	Europe	UK	mesic grassland	180	53.99	-2.63	8	1322	Stevens
marc.ar	South America	AR	grassland	6	-37.72	-57.42	13.9	838	Alberti/Daleo
mtca.au	Australia	AU	savanna	285	-31.78	117.61	17.3	330	Prober
podo.ec	South America	EC	paramo	3291	-4.11	-79.16	10.9	974	Báez

rook.uk	Europe	UK	mesic grassland	60	51.41	-0.64	9.8	706	Crawley
saline.us	North America	US	mixed grass prairie	440	39.05	-99.10	11.8	607	Smith/La Pierre
sevi.us	North America	US	desert grassland	1600	34.36	-106.69	12.6	252	Collins
sgs.us	North America	US	shortgrass prairie	1650	40.82	-104.77	8.4	365	Blumenthal/Brown/Klein
shps.us	North America	US	shrub steppe	910	44.24	-112.20	5.5	262	Adler
spin.us	North America	US	pasture	271.3	38.14	-84.50	12.5	1140	McCulley
temple.us	North America	US	tallgrass prairie	184	31.04	-97.35	19.1	871	Fay
ukul.za	Africa	ZA	mesic grassland	842.5	-29.67	30.40	18.1	880	Kirkman/Hagenah
valm.ch	Europe	CH	alpine grassland	2320	46.63	10.37	0.3	1098	Risch/Schuetz
yarra.au	Australia	AU	mesic grassland	19	-33.61	150.73	17.2	898	Power

Supplementary Table 2: Soil edaphic properties at our 30 globally distributed sites on six continents. Site, continent of the site location, soil organic C content (Corg; %), soil total N content (Ntot; %), soil C:N ratio, soil pH, soil sand content (sand; %), soil silt content (Silt; %), soil clay content (Clay; %), water holding capacity (WHC; vol%), and soil bulk density (BD, g cm^{-3}). Description of mean annual precipitation and temperature, elevation, grassland type and the coordinates of each site can be found in Supplementary Table 1.

Site	Continent	Corg	Ntot	C:N	pH	Sand	Silt	Clay	WHC	BD
bari.ar	South America	2.3	0.2	14.2	5.6	79.1	17.5	3.4	43.2	0.9
bldr.us	North America	0.9	0.1	11.7	5.7	73.2	15.1	11.8	28.6	1.4
bogong.au	Australia	6.1	0.4	14.7	3.8	71.2	13.2	15.7	49.6	0.8
burrawan.au	Australia	0.9	0.1	16.4	4.7	82.5	12.0	5.5	26.3	1.4
cbgb.us	North America	0.8	0.1	11.1	5.5	88.4	7.3	4.4	25.0	1.1
cdcr.us	North America	2.2	0.1	15.6	5.0	89.9	7.2	3.0	25.9	1.1
cdpt.us	North America	1.1	0.1	11.2	5.6	76.4	13.7	9.9	37.6	1.3
chilcas.ar	South America	4.0	0.4	10.9	5.5	48.2	42.5	9.3	42.1	0.8
comp.pt	Europe	1.2	0.1	13.8	4.4	79.8	15.6	4.6	24.7	1.4
cowi.ca	North America	5.7	0.4	13.0	4.9	58.7	23.6	17.7	33.5	0.6
frue.ch	Europe	3.5	0.4	9.8	4.9	44.8	33.9	21.4	44.5	1.0
jena.de	Europe	5.0	0.5	10.7	6.9	9.1	39.2	51.8	36.6	1.0
kibber.in	Asia	3.3	0.2	21.5	7.6	38.9	36.8	24.3	33.1	1.1
kilp.fi	Europe	7.8	0.6	13.5	3.9	59.8	28.5	11.7	57.0	0.6
koffler.ca	North America	2.6	0.2	11.1	6.9	62.8	27.9	9.4	30.7	1.0
konz.us	North America	3.9	0.3	14.3	5.6	15.6	49.4	35.0	43.2	0.9
lancaster.uk	Europe	22.3	1.3	17.8	4.1	70.6	6.9	22.5	63.8	0.5
marc.ar	South America	4.0	0.4	11.0	7.2	72.1	18.2	9.7	48.7	0.9
mtca.au	Australia	0.8	0.1	15.4	4.4	82.9	10.5	6.6	22.5	1.4
podo.ec	South America	7.5	0.4	19.0	3.3	50.8	36.3	12.9	56.0	0.4
rook.uk	Europe	3.2	0.3	12.3	3.4	83.3	10.7	6.0	41.0	1.1
saline.us	North America	4.1	0.3	15.1	6.7	26.8	44.3	28.9	35.2	1.1
sevi.us	North America	0.3	0.0	9.8	7.7	86.1	8.2	5.7	27.7	1.4
sgs.us	North America	1.1	0.1	10.7	5.1	72.6	15.2	12.2	37.7	1.2
shps.us	North America	2.5	0.2	13.1	7.5	50.5	34.7	14.9	44.7	1.2
spin.us	North America	2.2	0.2	9.1	5.6	14.8	56.7	28.6	43.1	1.1
temple.us	North America	10.0	0.4	23.6	7.3	21.1	25.6	53.3	44.5	0.7
ukul.za	Africa	5.1	0.3	16.1	5.1	12.5	35.8	51.7	39.9	0.9
valm.ch	Europe	4.5	0.3	13.3	4.9	68.0	22.4	9.6	37.7	0.9
yarra.au	Australia	0.9	0.1	11.4	4.5	80.1	15.6	4.3	29.6	1.2

Supplementary Table 3. Model selection results for realised and potential soil net N_{min} starting with the full model including all explanatory variables. Microbial biomass was replaced by soil organic C. Model selection criteria were set at delta AICc < 2 due to our small sample size. All results are based on linear mixed effect models with site identity as a random factor. Exp. vars. incl. = All explanatory variables included in the respective model, Estimate = parameter estimate, SE = parameter estimate standard error, p = p-value related to each variable, df = degrees of freedom of the component models, AICc = corrected Akaike's information criterion, T.q.wet = temperature of the wettest quarter, AOB = ammonia oxidising bacteria, Tvar = temperature seasonality, Organic C = soil organic C content, The total number of observations in all models = 85, the total number of sites in all models = 30.

**Top models**	**Exp. vars incl.**	**Estimate**	**SE**	**P**	**df**	**AICc**
		Realised soil net N_{min}				
Model 1	Intercept	0.521	0.041	<0.001	4	24.04
	Clay content	0.121	0.039	0.003		
Model 2	Intercept	0.518	0.033	<0.001	5	24.05
	T.q.wet	0.131	0.037	0.001		
	Organic C	0.140	0.035	<0.001		
Model 3	Intercept	0.518	0.036	<0.001	5	24.73
	T.q.wet	0.122	0.039	0.004		
	Bulk density	-0.133	0.036	<0.001		
		Potential soil net N_{min}				
Model 4	Intercept	0.587	0.042	<0.001	6	68.64
	AOB	0.123	0.039	0.003		
	T.q.wet	-0.194	0.045	<0.001		
	Tvar	0.125	0.045	0.010		
Model 5	Intercept	0.587	0.046	<0.001	5	69.29
	AOB	0.134	0.041	0.002		
	T.q.wet	-0.149	0.046	0.003		
Model 6	Intercept	0.589	0.052	<0.001	5	69.47
	T.q.wet	-0.206	0.056	0.001		
	Tvar	0.152	0.053	0.009		
Model 7	Intercept	0.590	0.057	<0.001	4	70.58
	T.q.wet	-0.150	0.058	0.015		

Supplementary Table 4. List of predictor variables used in the model selection process. MAT = Mean annual temperature, MAP = mean annual precipitation, Tvar = temperature seasonality, T = temperature, P = precipitation, AOA = archaeal ammonia oxidisers, AOB = bacterial ammonia oxidisers, log = transformed using log. "Variable name" according to Supplementary Figure 7. Variables in bold were retained to be used in our linear mixed effect models. For correlation among variables see Supplementary Figure 7 and Supplementary Table 5.

Group	Variable	Variable name
Climate	**MAT (°C)**	**bioclim.T.ann**
	**MAP (mm)**	**bioclim.P.ann**
	**Tvar (SE monthly means x 100)**	**bioclim.Tvar**
	T warmest quarter (°C)	bioclim.T.q.warm
	**T wettest quarter (°C)**	**bioclim.T.q.wet**
	T driest quarter (°C)	bioclim.T.q.dry
	P wettest quarter (mm)	bioclim.P.q.wet
Soil chemistry	**pH ($CaCl_2$)**	**ch.pH**
	**NO_3^- (mgkg)**	**ch.NO3start.mgkg**
	Total carbon (%)	ch.Ctot.perc.log
	Organic carbon (%)	ch.Corg.perc.log
	Total nitrogen (%)	ch.Ntot.perc.log
	**C:N ratio**	**ch.CtoN.log**
Soil texture	Pore space (vol%)	ch.porespace
	**Water holding capacity (vol%)**	**ch.WSK.vol**
	**Bulk density (g cm^{-3})**	**ch.bulkdensity.gcm3**
	**Clay content (%)**	**ch.clay.perc**
Soil biology	**AOA (archaeal amoA gene copies g^{-1} soil)**	**archaea.cpg.log**
	**AOB (bacterial amoA gene copies g^{-1} soil)**	**bacteria.cpg.log**
	**Microbial biomass (ug g^{-1} soil)**	**micr.biom.log**

Supplementary Table 5. Variables selected to be used in the linear mixed effect models (= left column). Variables that were highly related to a selected variable were dropped from the final dataset (= right column). Note that bulk density, organic C and microbial biomass are correlated, but were kept within the dataset (see methods for detailed explanation).

**Selected variable**	**Correlated with selected variable (r > |0.7|)**
MAT (°C)	T driest quarter, T warmest quarter
MAP (mm)	P wettest quarter
Tvar (SE monthly means x 100)	
T wettest quarter (°C)	T warmest quarter
pH ($CaCl_2$)	
NO_3^- (mgkg)	
C:N ratio	
Water holding capacity (vol%)	
Bulk density (g cm^{-3})	Total C, total N, pore space, organic C, microbial biomass
Clay content (%)	Sand content
AOA (archaeal amoA gene copies g^{-1} soil)	
AOB (bacterial amoA gene copies g^{-1} soil)	
Microbial biomass (ug g^{-1} soil)	Total C, total N, pore space, organic C, bulk density

Supplementary Table 6. Details of author contributions.

Name	Institution & Address	email	Developed and framed research question(s)	Ana-lysed samples	Ana-lysed data	Contri-buted to data analyses	Wrote paper	Contri-buted to paper writing	Site coor-dinator	Nutrient Network coordinator
Anita C. Risch	Swiss Federal Institute for Forest, Snow and Landscape Research, Zuercherstrasse 111, 8903 Birmensdorf, Switzerland	anita.risch@wsl.ch	x		x		x		x	
Stephan Zimmermann	Swiss Federal Institute for Forest, Snow and Landscape Research, Zuercherstrasse 111, 8903 Birmensdorf, Switzerland	stephan.zimmermann@wsl.ch	x	x				x		
Raul Ochoa-Hueso	Department of Biology, IVAGRO, University of Cádiz, Campus de Excelencia Internacional Agroalimentario (ceiA3), Campus Rio San Pedro, 11510 Puerto Real, Cádiz, Spain	rochoahueso@gmail.com			x			x		
Martin Schütz	Swiss Federal Institute for Forest, Snow and Landscape Research, Zuercherstrasse 111, 8903 Birmensdorf, Switzerland	martin.schuetz@wsl.ch	x			x		x	x	
Beat Frey	Swiss Federal Institute for Forest, Snow and Landscape Research, Zuercherstrasse 111, 8903 Birmensdorf, Switzerland	beat.frey@wsl.ch		x				x		
Jennifer Firn	Queensland University of Technology (QUT), School of Earth, Environmental and Biological Sciences, Science and Engineering Faculty, Brisbane, QLD, 4001 Australia.	jennifer.firn@qut.edu.au				x		x	x	
Philip A. Fay	USDA-ARS Grassland, Soil, and Water Research Laboratory, Temple, TX, 76502, USA	philip.fay@ars.usda.gov						x	x	
Frank Hagedorn	Swiss Federal Institute for Forest, Snow and Landscape Research,	frank.hagedorn@wsl.ch	x			x		x		

	Zuercherstrasse 111, 8903 Birmensdorf, Switzerland				
Elizabeth T. Borer	Department of Ecology, Evolution, and Behavior, University of Minnesota, St. Paul, MN 55108	borer@umn.edu	x	x	x
Eric W. Seabloom	Department of Ecology, Evolution, and Behavior, University of Minnesota, St. Paul, MN 55108, USA	seabloom@umn.edu	x	x	x
W. Stan Harpole	1) Department of Physiological Diversity, Helmholtz Center for Environmental Research – UFZ, Permoserstrasse 15, Leipzig 04318, Germany. 2) German Centre for Integrative Biodiversity Research (iDiv) Halle-Jena-Leipzig, Deutscher Platz 5e, Leipzig 04103, Germany. 3)Institute of Biology, M artin Luther University Halle-Wittenberg, Am Kirchtor 1, Halle (Saale) 06108, Germany.	stan.harpole@ufz.de	x	x	x
Johannes M.H. Knops	1) School of Biological Sciences, 211A Manter Hall, University of Nebraska, Lincoln NE 68588, USA, 2) Department of Health and Environmental Sciences, Xi'an Jiatong Liverpool University, Suzhou, 215213, China	jknops2@unl.edu	x	x	
Rebecca L. McCulley	Department of Plant and Soil Sciences, University of Kentucky, Lexington, KY 40546-0312, USA	rebecca.mcculley@uky.edu	x	x	
Arthur A.D. Broadbent	1) School of Earth and Environmental Sciences, Michael Smith Building, The University of Manchester, Oxford Road, Manchester, M13 9PT, UK; 2) Lancaster Environment Centre, Lancaster University, Lancaster, LA1 4YW, UK	broadbent.arthur@gmail.com	x		
Carly J. Stevens	Lancaster Environment Centre, Lancaster University, Lancaster, LA1 4YQ, UK	c.stevens@lancaster.ac.uk	x	x	

Maria L. Silveira	University of Florida, Range Cattle Research and Education Center. Ona, FL 33865, USA	mlas@ufl.edu		x	x
Peter B. Adler	Department of Wildland Resources and the Ecology Center, 5230 Old Main, Utah State University, Logan, UT 84103, USA	peter.adler@usu.edu	x	x	x
Selene V. Báez Jacome	Departamento de Biología, Escuela Politécnica Nacional del Ecuador, Ladrón de Guevera E11-253 y Andalucía, Quito, Ecuador.	selene.baez@epn.edu.ec		x	x
Lori A. Biederman	Department of Ecology, Evolution, and Organismal Biology, Iowa State University, Ames, IA 50011, USA	lbied@iastate.edu		x	x
John M. Blair	Division of Biology, Kansas State University, Manhattan, KS 66502, USA	jblair@ksu.edu		x	x
Cynthia S. Brown	Department of Bioagricultural Sciences and Pest Management, Graduate Degree Program in Ecology, Colorado State University, 1177 Campus Delivery, Fort Collins, Colorado, USA	cynthia.s.brown@colostate.edu		x	x
Maria C. Caldeira	Centro de Estudos Florestais, Instituto Superior de Agronomia, Universidade de Lisboa, Tapada da Ajuda, 1349-017 Lisboa, Portugal	mcaldeira@isa.ulisboa.pt		x	x
Scott L Collins	Department of Biology, University of New Mexico, Albuquerque, NM 87131 USA	scollins@unm.edu		x	x
Pedro Daleo	Instituto de Investigaciones Marinas y Costeras (IIMyC), Universidad Nacional de Mar del Plata, CONICET, Mar del Plata, Argentina	pdaleo@mdp.edu.ar		x	x
Agustina di Virgilio	INIBIOMA(CONICET-UNCOMA), Universidad Nacional del Comahue, Grupo de Investigaciones en Biología de la Conservación (GrINBic) Laboratorio Ecotono-, Quintral 1250, Bariloche, Argentina	adivirgilio@comahue-conicet.gob.ar		x	x

Anne Ebeling	Institute of Ecology and Evolution, University Jena, Dornburger Str. 159, 07743 Jena, Germany	anne.ebeling@uni-jena.de	x	x
Nico Eisenhauer	1) German Centre for Integrative Biodiversity Research (iDiv) Halle-Jena-Leipzig, Deutscher Platz 5e, 04103 Leipzig, Germany. 2) Institute of Biology, Leipzig University, Deutscher Platz 5e, 04103 Leipzig	nico.eisenhauer@idiv.de	x	x
Ellen Esch	University of California San Diego, 9500 Gilman Dr, La Jolla, CA 92037, USA	eesch@uoguelph.ca	x	x
Anu Eskelinen	1) Helmholtz Centre for Environmental Research UFZ, Leipzig, Germany, 2) German Centre for Integrative Biodiversity Research (iDiv) Halle-Jena-Leipzig, Leipzig, Germany, 3) Department of Ecology and Genetics, University of Oulu, Finland	anu.eskelinen@idiv.de	x	x
Nicole Hagenah	Mammal Research Institute, Department of Zoology and Entomology, University of Pretoria, Pretoria, South Africa	nicole.hagenah@up.ac.za	x	x
Yann Hautier	Ecology and Biodiversity Group, Department of Biology, Utrecht University, Padualaan 8, 3584 CH Utrecht, The Netherlands.	y.hautier@uu.nl	x	x
Kevin P. Kirkman	University of KwaZulu-Natal, Pietermaritzburg, Private Bag X01, Scottsville 3209, South Africa	kirkmank@ukzn.ac.za	x	x
Andrew S. MacDougall	University of Guelph, Department of Integrative Biology, Guelph, Ontario Canada N1G 2W1	amacdo02@uoguelph.ca	x	x
Joslin L. Moore	School of Biological Sciences, Monash University, Claytion VIC 3800, Australia	joslin.moore@monash.edu	x	x
Sally A. Power	Hawkesbury Institute for the Environment, Western Sydney University, Locked Bag 1797, Penrith, NSW 2751, Australia.	s.power@westernsydney.edu.au	x	x

Suzanne M. Prober	CSIRO Land and Water, Private Bag 5, Wembley WA 6913 Australia	suzanne.prober@csiro.au		x	x
Christiane Roscher	1) UFZ, Helmholtz Centre for Environmental Research, Physiological Diversity, Permoserstrasse 15, 04318 Leipzig, Germany, 2) German Centre for Integrative Biodiversity Research (iDiv) Halle-Jena-Leipzig, Deutscher Platz 5e, 04103 Leipzig, Germany	christiane.roscher@ufz.de		x	x
Mahesh Sankaran	1) National Centre for Biological Sciences, TIFR, Bengaluru 560065, India<br>2) School of Biology, University of Leeds, Leeds LS2 9JT, UK	mahesh@ncbs.res.in		x	x
Julia Siebert	1) German Centre for Integrative Biodiversity Research (iDiv) Halle-Jena-Leipzig, Deutscher Platz 5e, 04103 Leipzig, Germany. 2) Institute of Biology, Leipzig University, Deutscher Platz 5e, 04103 Leipzig	julia.siebert@idiv.de	x	x	
Karina L. Speziale	INIBIOMA (CONICET-UNCOMA), Universidad Nacional del Comahue, Grupo de Investigaciones en Biología de la Conservación (GrInBiC) Laboratorio Ecotono, Quintral 1250, Bariloche, Argentina	karina.speziale@comahue-conicet.gob.ar		x	x
Pedro Tognetti	Universidad de Buenos Aires. Facultad de Agronomía. Instituto de Investigaciones Fisiológicas y Ecológicas vinculadas a la Agricultura (IFEVA). CONICET, Buenos Aires, Argentina.	tognetti@agro.uba.ar		x	x
Risto Virtanen	1) Helmholtz Centre for Environmental Research UFZ, Leipzig, Germany, 2) German Centre for Integrative Biodiversity Research (iDiv) Halle-Jena-Leipzig, Leipzig, Germany, 3) Department	risto.virtanen@oulu.fi		x	x

	of Ecology and Genetics, University of Oulu, Finland					
Laura Yahdjian	Universidad de Buenos Aires. Facultad de Agronomía. Instituto de Investigaciones Fisiológicas y Ecológicas vinculadas a la Agricultura (IFEVA). CONICET, Buenos Aires, Argentina.	yahdjian@agro.uba.ar			x	x
Barbara Moser	Swiss Federal Institute for Forest, Snow and Landscape Research, Zuercherstrasse 111, 8903 Birmensdorf, Switzerland	barbara.moser@wsl.ch	x	x	x	
