# Supplementary material for: Soil net nitrogen mineralisation across global grasslands
Source: Nat Commun. 2019 Oct 31;10:4981. doi: 10.1038/s41467-019-12948-2 (PMC6823350; doi:10.1038/s41467-019-12948-2)
Supplement: Supplementary file 3 — Reporting Summary [file 41467_2019_12948_MOESM3_ESM.pdf]

nature research

Corresponding author(s): *Double-blind peer review submissions: write DBPR and your manuscript number here instead of author names.*

Last updated by author(s): *YYYY-MM-DD*

Reporting Summary

Nature Research wishes to improve the reproducibility of the work that we publish. This form provides structure for consistency and transparency in reporting. For further information on Nature Research policies, see Authors & Referees and the Editorial Policy Checklist.

Statistics

For all statistical analyses, confirm that the following items are present in the figure legend, table legend, main text, or Methods section.

n/a	Confirmed	
☐	☒	The exact sample size (*n*) for each experimental group/condition, given as a discrete number and unit of measurement
☐	☒	A statement on whether measurements were taken from distinct samples or whether the same sample was measured repeatedly
☐	☒	The statistical test(s) used AND whether they are one- or two-sided<br>*Only common tests should be described solely by name; describe more complex techniques in the Methods section.*
☐	☒	A description of all covariates tested
☐	☒	A description of any assumptions or corrections, such as tests of normality and adjustment for multiple comparisons
☐	☒	A full description of the statistical parameters including central tendency (e.g. means) or other basic estimates (e.g. regression coefficient) AND variation (e.g. standard deviation) or associated estimates of uncertainty (e.g. confidence intervals)
☐	☒	For null hypothesis testing, the test statistic (e.g. *F*, *t*, *r*) with confidence intervals, effect sizes, degrees of freedom and *P* value noted<br>*Give P values as exact values whenever suitable.*
☒	☐	For Bayesian analysis, information on the choice of priors and Markov chain Monte Carlo settings
☐	☒	For hierarchical and complex designs, identification of the appropriate level for tests and full reporting of outcomes
☒	☐	Estimates of effect sizes (e.g. Cohen's *d*, Pearson's *r*), indicating how they were calculated

Our web collection on statistics for biologists contains articles on many of the points above.

Software and code

Policy information about availability of computer code

Data collection	N/A
Data analysis	All analyses was conducted with R 3.4.4 (for LMM) and R 3.4.0 for q-sep SEM. Packages used were nlme (version 3.131.1), MuMin (version 1.42.1), piecewiseSEM (version 2.0.2)

For manuscripts utilizing custom algorithms or software that are central to the research but not yet described in published literature, software must be made available to editors/reviewers. We strongly encourage code deposition in a community repository (e.g. GitHub). See the Nature Research guidelines for submitting code & software for further information.

Data

Policy information about availability of data

All manuscripts must include a data availability statement. This statement should provide the following information, where applicable:

- Accession codes, unique identifiers, or web links for publicly available datasets
- A list of figures that have associated raw data
- A description of any restrictions on data availability

The data is available at www.envidat.ch. doi: 10.16904/envidat.87; Data for Figures 2, 4, 5 and Supplementary Figures 1 to 8 can be found in the source data file.

Field-specific reporting

Please select the one below that is the best fit for your research. If you are not sure, read the appropriate sections before making your selection.

☐ Life sciences ☐ Behavioural & social sciences ☒ Ecological, evolutionary & environmental sciences

Ecological, evolutionary & environmental sciences study design

All studies must disclose on these points even when the disclosure is negative.

Study description	The 30 study sites are part of the Nutrient Network Global Research Cooperative (NutNet, https://nutnet.umn.edu/). At each site the effects of nutrient addition and herbivore exclusion treatments are examined via a random-block design. This block design is replicated three times at the majority of the sites. Here, for four sites, we only had data from one (1 site) and two blocks, respectively. This study is restricted to data collected from the untreated control plots. Each plot has a size of 5 m x 5 m and is divided into four 2.5 m x 2.5 m subplots. Each subplot is further divided into four 1 m x 1 m square sampling plots, one of which is set aside for soil sampling28. Plots are separated by at least 1 m wide walkways. Mean annual temperature of our sites ranged from -4 to 22 °C, mean annual precipitation from 252 mm to 1592 mm, and elevations from 6 m to 4261 m above sea level (Fig 2, Supplementary Table S1). Soil organic C varied from 0.32 to 22.30%, soil total N from 0.03 to 1.25% and the soil C to N ratio from 9.07 to 23.64 between our 30 sites. Also soil clay content (3.0 to 53.3%) and soil pH (3.25 to 7.71) span a large gradient across our 30 sites (Supplementary Table S2). Thus, our 30 sites cover a wide range of grasslands globally that are typical for the respective region.
Research sample	We collected a total of 4 soil cores per sampling plot (1 x 1 m). One core was used for on-site incubation (realised soil N mineralisation), two were composited, mixed and used for assessing soil chemical and biological properties as well as potential soil N mineralisation. One was used to assess soil physical properties.
Sampling strategy	The number of samples was chosen to allow for analyses of all data, but to minimize the efforts for sampling and shipping. We have have several replicates per site to capture additional varability in soil properties.
Data collection	Each site received an identical package shipped from the Swiss Federal Institute for Forest, Snow and Landscape Research (WSL) with material to be used for sampling and incubations (steel cores and rings, resin bags, caps, gloves, etc.). For the field incubation we followed the protocol by Risch et al. 2015. Briefly, at randomised locations in each plot we clipped the vegetation and then we drove a 5 x 15 cm (diameter x depth) steel cylinder 13.5 cm deep into the soil so that 1.5 cm on top of the cylinder remained empty. To capture incoming N from run-off and/or deposition we placed a polyester mesh bag (mesh-size 250 µm) filled with 13.2±0.9 g of acidic and alkaline exchanger resin (1:1 mixture; ion-exchanger I KA/ion-exchanger III AA, Merck AG, Darmstadt) into the upper 1.5 cm space of the cylinder. The bag was fixed in place with a metal Seeger ring (Bruetsch-Rüegger Holding, Urdorf, Switzerland). Thereafter we removed 1.5 cm soil at the bottom of the cylinder and placed another resin bag into the cylinder to capture N leached from the soil column. We made sure that the exchange resin was saturated with H+ and Cl- prior to filling the bags by stirring the mixture for 1 hour in HCl 1.2 M and then rinsing it with demineralised water until the electrical conductivity of the water reached 5 µS/cm. The cylinders including the resin bags were then re-inserted into the soil, at the same location where the sample was collected, flush with the soil surface, and incubated for 42 days (range 36 to 57). Each site chose the timing of incubation so that it started six weeks prior to peak plant biomass production. All the incubations were completed between February 2015 to January 2016. We additionally collected two soil cores of 5 cm diameter and 12 cm depth with a steel core at each sampling plot for potential soil net Nmin, soil chemical and biological analyses. We composited the two samples and then re-used the steel cylinder to collect one additional sample (5 x 12 cm) to assess soil physical properties. This third sample remained within the steel core and both ends were tightly closed with plastic caps. The capped steel cores were then gently packed to avoid further disturbance and together with the composited soil samples overnight-shipped to the laboratory at WSL.
Timing and spatial scale	Each site chose the timing of incubation so that it started six weeks prior to peak plant biomass production. All the incubations were completed between February 2015 to January 2016. Each team had to visit the site twice. Once to collect the samples and incubate the cores for realised soil net N mineralisation in the field, then to recollect the incubated cores after 6 weeks.
Data exclusions	For some sites we do not have 3 replicates as they either only had 2 blocks in the field (bldr.us) or we do not have potential mineralistation due to an accident in the laboratory (1 for lancaster.uk and 1 for podo.ec, 2 for cdcr.us).
Reproducibility	All samples were analysed in the same laboratory by the same people to assure quality control. Samples could be re-collected as all the sites are still active.
Randomization	Each site set up a random block design for the experiment. Soil samples were randomly collected within the 1 x 1 m sampling plots. Of the composited sample for soil chemical and biological properties and potential N mineralisation random sub-samples were taken.
Blinding	Our data was analysed using all replicates per site, but without knowing which site had which influence on the overall results.

Did the study involve field work? ☒ Yes ☐ No

Field work, collection and transport

Field conditions	Each site chose the timing of incubation so that it started six weeks prior to peak plant biomass production.
Location	Ours sites were distributed over 6 continents and 12 countries. Site name, continent, country, grassland type, elevation, latitude, longitude, mean annual temperature at the site and mean annual precipitation at the site can be found below.<br><br>Site Continent Country Grassland type Elevation Latitude Longitude MAT MAP<br>bari.ar South America AR grassland steppe 786 -41.01 -71.15 8.6 862<br>bldr.us North America US shortgrass prairie 1633 39.97 -105.23 9.7 425<br>bogong.au Australia AU alpine grassland 1760 -36.87 147.25 5.7 1592

burrawan.au Australia AU semiarid grassland 425 -27.73 151.14 18.4 683
cbgb.us North America US tallgrass prairie 275 41.79 -93.39 9 855
cdcr.us North America US tallgrass prairie 270 45.43 -93.21 6.3 750
cdpt.us North America US shortgrass prairie 965 41.20 -101.63 9.5 445
chilcas.ar South America AR mesic grassland 15 -36.28 -58.27 15.1 925
comp.pt Europe PT annual grassland 200 38.00 -8.00 16.5 554
cowi.ca North America CA old field 50 48.46 -123.38 9.8 764
frue.ch Europe CH pasture 995 47.11 8.54 6.5 1355
jena.de Europe DE grassland 320 50.93 11.53 8 610
kibber.in Asia IN alpine grassland 4241 32.32 78.01 1.1 504
kilp.fi Europe FI tundra grassland 700 69.05 20.83 -4.1 551
koffler.ca North America CA pasture 301 44.02 -79.54 6.4 815
konz.us North America US tallgrass prairie 440 39.07 -96.58 11.9 877
lancaster.uk Europe UK mesic grassland 180 53.99 -2.63 8 1322
marc.ar South America AR grassland 6 -37.72 -57.42 13.9 838
mtca.au Australia AU savanna 285 -31.78 117.61 17.3 330
podo.ec South America EC paramo 3291 -4.11 -79.16 10.9 974
rook.uk Europe UK mesic grassland 60 51.41 -0.64 9.8 706
saline.us North America US mixed grass prairie 440 39.05 -99.10 11.8 607
sevi.us North America US desert grassland 1600 34.36 -106.69 12.6 252
sgs.us North America US shortgrass prairie 1650 40.82 -104.77 8.4 365
shps.us North America US shrub steppe 910 44.24 -112.20 5.5 262
spin.us North America US pasture 271.3 38.14 -84.50 12.5 1140
temple.us North America US tallgrass prairie 184 31.04 -97.35 19.1 871
ukul.za Africa ZA mesic grassland 842.5 -29.67 30.40 18.1 880
valm.ch Europe CH alpine grassland 2320 46.63 10.37 0.3 1098
yarra.au Australia AU mesic grassland 19 -33.61 150.73 17.2 898

Access and import/export

Each site shipped soil samples to Switzerland twice. For each site the Swiss Federal Institute for Forest, Snow and Landscape Research WSL obtained an import permit from the Federal Office for Agriculture FOAG. Samples were stored and treated as required by the permit.

Disturbance

At each site walkways were established to minimize trampling efforts on the sampling plots

Reporting for specific materials, systems and methods

We require information from authors about some types of materials, experimental systems and methods used in many studies. Here, indicate whether each material, system or method listed is relevant to your study. If you are not sure if a list item applies to your research, read the appropriate section before selecting a response.

Materials & experimental systems

n/a	Involved in the study
☒	☐ Antibodies
☒	☐ Eukaryotic cell lines
☒	☐ Palaeontology
☒	☐ Animals and other organisms
☒	☐ Human research participants
☒	☐ Clinical data

Methods

n/a	Involved in the study
☒	☐ ChIP-seq
☒	☐ Flow cytometry
☒	☐ MRI-based neuroimaging
